# The Impact of intra-abdominal Pressure on Perioperative Outcomes in Robotic-Assisted Radical Prostatectomy: A Systematic Review and Network Meta-Analysis of Randomized Controlled Trials

**DOI:** 10.1155/2022/4974027

**Published:** 2022-11-14

**Authors:** Yuan Yang, Yushan Duan, Xiaohong Wan, Linjun Wan, Gang Wang, Jianlin Shao

**Affiliations:** ^1^Department of Anesthesiology, The First Affiliated Hospital, Kunming Medical University, Kunming, China; ^2^Department of Critical Care Medicine, The Second Affiliated Hospital, Kunming Medical University, Kunming, China

## Abstract

**Objective:**

The aim of the study is to analyze the impact of intra-abdominal pressure (IAP) on perioperative outcomes in robotic-assisted radical prostatectomy (RARP).

**Methods:**

We searched the PubMed, Cochrane Library, Science, Embase, and CNKI databases systematically, and the retrieval date was from the inception of the databases to April 2022. Randomized controlled trials on high intraabdominal pressure (HIAP) and low intraabdominal pressure (LIAP) in RARP were included. The meta-analysis was performed using Review Manager software (version 5.3).

**Results:**

Six studies involving 2,271 patients were included in the meta-analysis. Compared with patients who experienced HIAP, those who experienced LIAP had a lower incidence of postoperative ileus (POI) (risk ratio (RR): 0.42; 95% confidence interval (CI): 0.24 to 0.72; *p* = 0.002). However, there were no significant differences in hematoma (RR 2.22; 95% CI, 0.61 to 8.15; *p* = 0.23), positive margin rate (RR, 1.06; 95% CI, 0.84 to 1.32; *p* = 0.64), urinary retention (RR, 0.99; 95% CI, 0.51 to 1.94; *p* = 0.98), operative time (mean difference (MD), −0.36; 95% CI, −12.24 to 6.12; *p* = 0.51), or intraoperative blood loss (MD, −21.80; 95% CI, −55.28 to 11.68; *p* = 0.20) among patients undergoing LIAP and HIAP.

**Conclusion:**

Our study of published trials indicates that using LIAP during RARP may reduce the incidence of POI, and there were no differences in terms of hematoma, positive margin rate, urinary retention, operative time, or intraoperative blood loss.

## 1. Introduction

Robot-assisted radical prostatectomy (RARP) has become the gold standard for localized prostate cancer treatment since its inception in the 20th century [1, 2]. The improved vision provided by the robotic system is combined with a better understanding of the surgical anatomy, allowing improved surgical techniques to optimize postoperative recovery[3]. RARP accounts for more than 85% of prostatectomies performed in the United States [2]. The consistently reported benefits of RARP over open prostatectomy include a shorter duration of hospitalization and lower blood loss [4–6].

Over the past two decades, many studies on optimal intraabdominal pressure (IAP) for laparoscopy have emerged [7, 8]. However, studies on the impact of pneumoperitoneum on RARP are limited. The establishment of laparoscopic pneumoperitoneum can improve visualization, shorten operation time, and reduce blood loss [9]. However, abdominal insufflation of carbon dioxide may cause many physiological changes, such as reduced cardiac output, increased peak airway pressure, oliguria, and systemic acidosis [10–14]. As study shows, laparoscopic cholecystectomy with lower pneumoperitoneum pressure can reduce hospital stay and postoperative pain [15]. However, using lower pneumoperitoneum pressures may limit visualization, prolong the operative time, increase blood loss, or cause unintended damage to organs [7].

Available evidence from currently published studies that can inform clinical practice includes a retrospective study in 2018 [16] and two randomized controlled trials (RCTs) published in 2020 [17] and 2021 [18]. These studies suggest that low IAP during RARP is associated with a significant reduction in the incidence of postoperative bowel obstruction compared with standard IAP [16–18]. However, the impact of low IAP on operative time, length of hospital stay, and other surgical outcomes is unknown. In addition, a meta-analysis published around the same time [19] concluded that the use of low IAP (LIAP) during laparoscopic cholecystectomy reduced postoperative pain, including shoulder pain and length of hospital stay, compared with standard IAP (defined as 12–14 mmHg). Critically, no published meta-analyses have compared the impact of high IAP (HIAP) and LIAP on perioperative outcomes in RARP. Given these data gaps and the availability of existing studies, we conducted a meta-analysis to discuss the impact of IAP on perioperative outcomes in RARP.

## 2. Materials and Methods

### 2.1. Search Strategy

We systematically searched the PubMed, Cochrane Library, Science, Embase, and CNKI databases, and the retrieval date was from the database inception to April 2022. We searched the following terms: “prostate neoplasms,” “prostate cancer,” “prostatectomy,” “prostatectomies,” “suprapubic prostatectomy,” “retropubic prostatectomy,” “pneumoperitoneum,” “insufflation pressure,” “abdominal pressure,” and “pneumoperitoneum pressure.” Search strategies were formulated for different databases. A manual search of the references of articles related to the topic was performed to broaden the scope of the search. All the included studies were independently evaluated by two reviewers (Y. D. and Y. Y.), and all differences were resolved through discussion.

### 2.2. Inclusion/Exclusion Criteria

Following the PICOS principle, inclusion criteria were as follows: (1) studies were performed in adults diagnosed with prostate cancer; (2) included patients who received RARP; (3) compared different pneumoperitoneum pressures; (4) full papers containing at least one outcome parameter, such as the occurrence of postoperative ileus (POI), operative time, blood loss, positive margin, and so on; and (5) had an RCT or cohort study design. Exclusion criteria were as follows: non-RCTs, studies with incomplete or unavailable data, animal studies, systematic reviews, and reviews or republished studies.

### 2.3. Data Extraction

We extracted the following data from the studies in the meta-analysis: author, publication year, study design, basic information, pneumoperitoneum pressure, operative time, blood loss, positive margin, and occurrence of POI, hematoma, and urinary retention. When there was a continuous variable, the average value and standard deviation were calculated.

### 2.4. Quality Assessment

**Figure 1:**
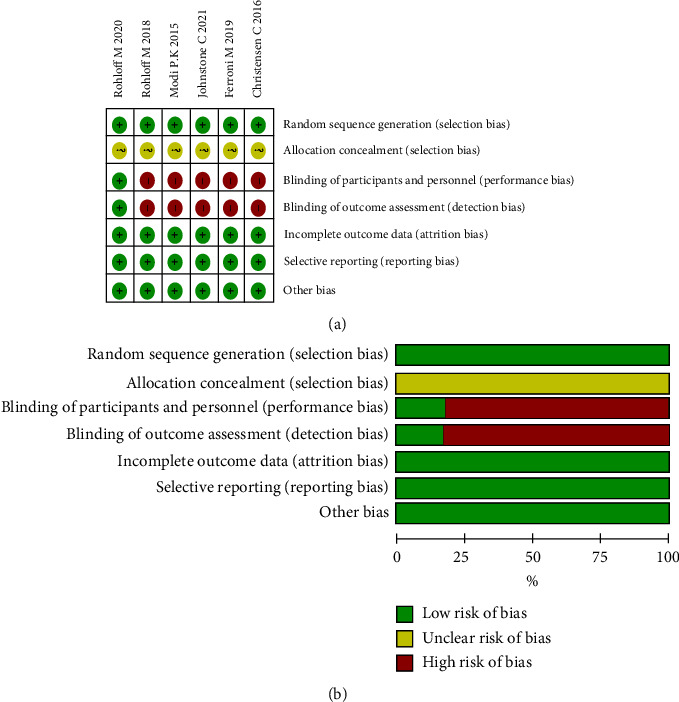
The risk of bias assessment for each trial using the risk of bias in Cochrane systematic reviews. (a) Risk of bias summary. (b) Risk of bias graph.

According to the recommendations of evidence-based medicine research guidelines, the risk of bias in Cochrane systematic reviews was used to evaluate the literature quality [20, 21]. The quality of the included studies was assessed using six indicators: randomization method, concealment of allocation scheme, blinding, completeness of outcome data, selective reporting of study results, and other sources of bias. Regarding the classification of quality assessments: studies with ≥5 items were considered to have a low risk of bias; 3-4 items, a moderate risk of bias; and <3 items, a high risk of bias. One article had a low risk of bias, and the other five articles all had a moderate risk of bias, all articles were of high quality. In Figure1(a), the standard is “+,” and the non-compliance is “−.” Figure 1(b) shows the proportion of each item in the methodological assessment.

### 2.5. Statistical Analysis

A meta-analysis of the included studies was performed using Review Manager version 5.3. For dichotomous variables, the risk ratio (RR) and 95% confidence interval (CI) were used as efficacy indicators for statistical analysis. Heterogeneity between studies was assessed using the *Q*-test and *I*^2^-test. If *p* < 0.05 or *I*^2^>50%, heterogeneity was considered to exist, and the random-effects model was used to combine the data; however, if (*p* ≥ 0.05 or *I*^2^ ＜ 50%), no heterogeneity was considered to exist, and data were combined using a fixed-effects model. *p* < 0.05 indicated that differences were statistically significant. A funnel plot was drawn to analyze potential publication bias.

### 2.6. Registration

The study was registered on PROSPERO (CRD42022351780).

## 3. Results

### 3.1. Study Characteristics

**Figure 2:**
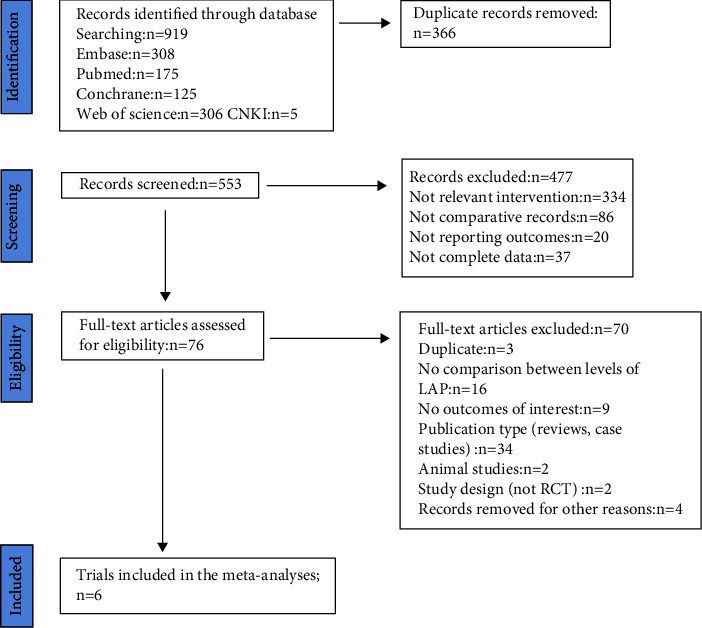
Flow diagram of studies identified, included, and excluded.

A total of 919 articles were initially retrieved. However, 843 articles were excluded because there were duplicates or irrelevant to our study. After reading the full text, another 70 articles were excluded. Finally, six studies with 2271 patients were included in our meta-analysis [16, 17, 22–25] (Figure 2). The characteristics of the included studies are shown in Table 1. The risk of bias in the Cochrane systematic reviews was used to evaluate the quality of the studies. All studies had a risk of bias, but most were moderate, and the average quality of each study was good. The results are shown in Figure 1.

### 3.2. Demographic Variables

Demographic variables were analyzed according to the included studies for each outcome parameter. There were no statistically significant differences between the demographic variables of the included studies (Table 2).

### 3.3. Postoperative Ileus

**Figure 3:**
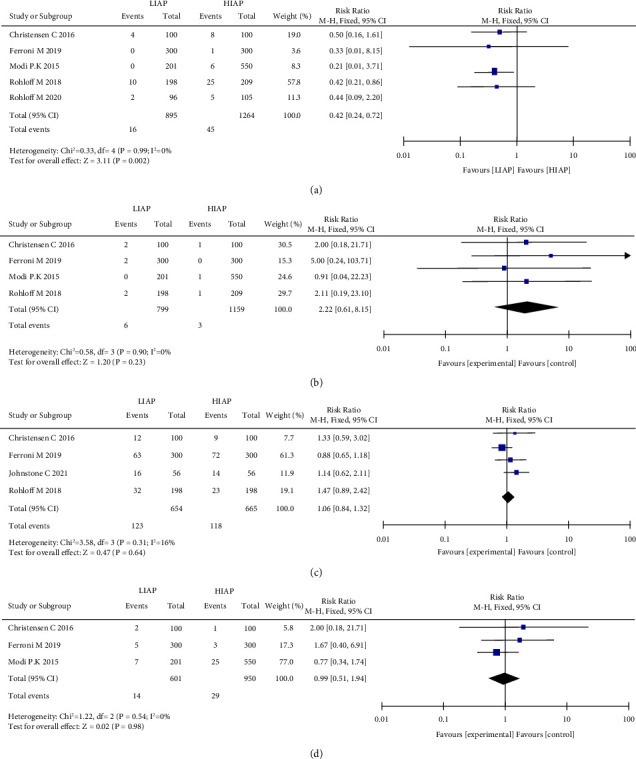
Forest plot and meta-analysis of postoperative ileus (a), hematoma (b), positive margin rate (c), and urinary retention (d).

Five articles were analyzed regarding POI [16, 17, 22–24]. A total of 2159 patients were included in the studies, of whom 895 experienced LIAP and 1264 experienced HIAP (Figure 3(a)). Compared with patients who experienced HIAP, patients with LIAP had a lower incidence of POI (fixed-effects model: RR, 0.42; 95% CI, 0.24 to 0.72; *p*=0.002; *I*^2^ = 0%).

### 3.4. Hematoma

Four articles were analyzed with respect to hematoma [16, 22–24]. A total of 1958 patients were included in the studies, of whom 799 experienced LIAP and 1159 experienced HIAP (Figure 3(b)). Hematoma was similar between the two groups, and no heterogeneity was observed (fixed-effects model: RR, 2.22; 95% CI, 0.61 to 8.15; *p* = 0.23; *I*^2^ = 0%).

### 3.5. Positive Margin Rate

For positive margin rate, four articles were analyzed [16, 22, 24, 25]. A total of 1319 patients were included in the studies; of which, 654 experienced LIAP and 665 experienced HIAP (Figure 3(c)). The positive margin rate was similar between the two groups, and no heterogeneity was observed (fixed-effects model: RR, 1.06; 95% CI, 0.84 to 1.32; *p*=0.64; *I*^2^ = 16%).

### 3.6. Urinary Retention

Three articles were analyzed regarding urinary retention [22–24]. A total of 1551 patients were included in the studies; of which, 601 experienced LIAP and 950 experienced HIAP (Figure 3(d)). The urinary retention rate was similar between the two groups, and no heterogeneity was observed (fixed-effects model: RR, 0.99; 95% CI, 0.51 to 1.94; *p*=0.98; *I*^2^ = 0%).

### 3.7. Operative Time

**Figure 4:**
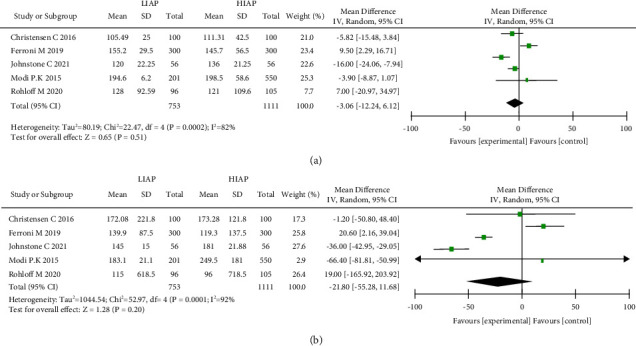
Forest plot and meta-analysis of operative time (a) and intraoperative blood loss (b).

Five studies were analyzed concerning operative time [17, 22–25]. A total of 1864 patients were included in the studies; 753 experienced LIAP and 1111 experienced HIAP. Because the heterogeneity was considerable (*I*^2^ = 82%), we used a random-effects model (Figure 4(a)). The final results showed no significant difference between the two groups (random-effects model: MD, −0.36; 95% CI, −12.24 to 6.12; *p*=0.51; *I*^2^ = 82%).

### 3.8. Intraoperative Blood Loss

Five studies on intraoperative blood loss were conducted [17, 22–25], and they included 1864 patients, with 753 experiencing LIAP and 1111 experiencing HIAP. Because heterogeneity was considerable (*I*^2^ = 92%), we used a random-effects model (Figure 4(b)). The final results showed no statistically significant difference between the two groups (random-effects model: MD, −21.80; 95% CI, −55.28 to 11.68; *p*=0.20; *I*^2^ = 92%).

### 3.9. Publication Bias

**Figure 5:**
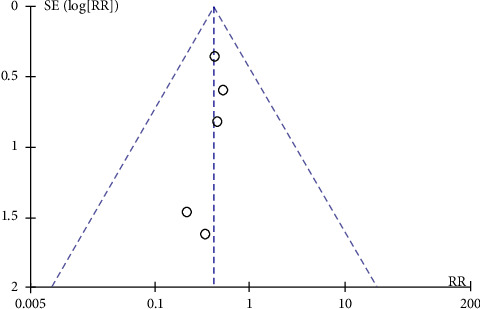
Funnel plot.

A funnel plot test was performed on the included studies. The results showed that the scattered points were mostly distributed in the middle and upper parts of the funnel, indicating that the research precision was high. However, the distribution was skewed and the distribution on both sides was asymmetric, indicating that the study had a certain degree of publication bias (Figure 5).

## 4. Discussion

RARP is the gold standard treatment for localized prostate cancer treatment [1, 2]. As study shows, laparoscopic cholecystectomy with lower pneumoperitoneum pressure can reduce hospital stay and postoperative pain [15]. However, the use of lower pneumoperitoneum pressures has many limitations. Therefore, we conducted a meta-analysis to discuss the impact of IAP on the perioperative outcomes of RARP.

Postoperative parameters are important in assessing the safety of surgery, and the postoperative parameters assessed in our study included POI, hematoma, positive margin rate, and urinary retention. Our meta-analysis focused on assessing whether low-pressure pneumoperitoneum could reduce the incidence of POI. Regarding the incidence of POI, our study showed that there was a significant difference between the HIAP and LIAP groups, which is consistent with the results of most studies; the incidence of POI in the LIAP group was significantly lower than that in the HIAP group. The incidence of POI ranges from 3% to 10% in urological procedures, with approximately $1.5 billion annually in U.S. health care costs [26, 27]. Schilling et al. found that increasing the pneumoperitoneum pressure from 10 mmHg to 15 mmHg reduced the blood flow to the jejunum and colon by 32% and 44%, respectively [28]. During laparoscopic surgery, the compression of intestinal gas may lead to decreased mesenteric blood flow and decreased intestinal motility, which may lead to prolonged recovery time of intestinal function, possibly contributing to the development of POI [29]. However, interestingly, Rohloff et al. found that two other independent variables that contributed to the increased risk of POI, smoking, and maintenance IV fluids were independently associated with a higher incidence of POI [17]. Although these variables are unlikely to be the direct cause of POI, it is important to consider these factors.

Other postoperative parameters, included hematoma formation, positive margin rate, and urinary retention. Our study showed that there was no statistically significant difference between the HIAP group and the LIAP group. These secondary results demonstrated noninferiority in the LIAP group, indicating that the use of lower pneumoperitoneum pressures is safe when performing RARP. However, because of the limited data available in the study, and the low event rates for some outcomes, the ability to detect a significant difference may be difficult even if different IAP levels had a significant effect on these outcomes.

Another major problem of low-pressure pneumoperitoneum is the assessment of the intraoperative parameters. In terms of operative time and intraoperative blood loss, our study showed that there was no statistically significant difference between the HIAP group and the LIAP group. Surgeons are concerned that the use of lower pneumoperitoneum pressures may limit visualization and lead to blood loss, increased operative time, and unintended damage to organs and structures. Christensen et al. found that there was a statistically significant increase of 10.5 minutes in the mean operating time among patients in the 6 mmHg group (145.7 vs. 155.2 min; *p* < 0.001) and a 20 mL increase in estimated blood loss (119.3 vs. 139.9 mL; *p* < 0.001) [22]. However, Johnstone et al. found that the operative time was 136.5 minutes (120–195) in the HIAP group and 120 minutes (106–145) in the LIAP group. Despite the use of lower pneumoperitoneum pressures, the LIAP group had a shorter operative time, although the difference was not statistically significant (*p*=0.0525). At the same time, intraoperative blood loss in the LIAP group was significantly reduced, with an average blood loss of 35 mL less than that in the HIAP group (145 vs. 181 mL; *p*=0.0029) [25]. The reason for this may be the different experiences of doctors in different institutions, and the difference in the operation may lead to deviations in the experimental results.

Typically, 15 mmHg is the standard pneumoperitoneum pressure used during surgery. In our meta-analysis, four of the included studies used the standard pneumoperitoneum pressure as the HIAP group. However, the disadvantage is that in the study by Modi et al., 15 mmHg was used as the low pneumoperitoneum pressure group [23]. The research results of Modi et al. showed that 17 cases (8.46%) of complications were found in the control group and 47 cases (8.55%) in the experimental group; the difference in the incidence of complications between the groups was not statistically significant. This suggests that the use of 20 mmHg pressure in RARP is safe and that increased inflation pressure is not associated with higher complication rates. However, the incidence of POI in the HIAP group was still significantly higher than that in the LIAP group (6 vs. 0), which is consistent with the results of our meta-analysis. At the same time, combined with the results of this study, it is also proven that lower pneumoperitoneum pressure is not inferior to higher pneumoperitoneum pressure. In the study by Rohloff et al., 12 mmHg was used as the high pneumoperitoneum pressure group, and the primary endpoint of the study was the occurrence of POI. The study showed that seven patients experienced POI, including five (4.8%) in the HIAP group and two (2%) in the LIAP group [17]. Unfortunately, the findings were not statistically significant but could demonstrate that lowering pneumoperitoneum pressure may reduce the incidence of POI. Therefore, the lack of standardized IAP may lead to some inconsistency between the results of RCT in the literature. Therefore, to reduce the resulting analytical error, our analysis was based only on evidence from RCTs. Then, we only included studies with RARP-specific data to improve homogeneity and comparability.

We rigorously completed this meta-analysis under the guidance of PRISMA [16], but our analysis still has some certain limitations. First, most of the included studies adopted a retrospective study design, only six articles were included, and some studies had small sample sizes and low levels of evidence. Second, the number of clinical studies included in the evaluation of various indicators was limited, and it was difficult to obtain valid evidence. Also, the number of clinical studies included in the evaluation of various indicators is limited and it is difficult to obtain valid evidence. Third, the lack of standardized IAPs may have contributed to some inconsistencies between the results of RCT studies in the literature. Fourth, differences in surgeon skills, hospital protocols, and hospital resources may have contributed to the observed heterogeneity in some measures. Nonetheless, our meta-analysis included high-level evidence, some of the included studies were prospective studies, most of the included studies were published in the past five years, and outcome indicators are well documented, which greatly increase the credibility of our results.

## 5. Conclusion

Our study of published trials indicates that using LIAP during RARP may reduce the incidence of POI, and there were no differences in terms of hematoma, positive margin rate, urinary retention, operative time, or intraoperative blood loss.

## 6. Disclosure

Yuan Yang and Yushan Duan are co-first authors.

## Figures and Tables

**Table 1:** Baseline characteristics of include studies and methodological assessment.

Author and year	Study design	Study arm based on proposed IAP categorization	Study arm (mmHg)	Patients (*n*)	BMI (kg/m^2^)	Age (years)	Prostate weight (g)	Preoperative PSA (ng/ml)
Rohloff (2020)	Prospective	LIAP vs. HIAP	8 vs. 12	96/105	NA	NA	62 ± 133.3	NA
							55 ± 149.6	

Rohloff (2018)	Retrospective	LIAP vs. HIAP	12 vs. 15	198/209	29.88 ± 6.5	NA	NA	NA
					29.66 ± 7.25			

Johnstone (2021)	Prospective	LIAP vs. HIAP	6 vs. 13–15	56/56	NA	NA	48 ± 20	NA
							46.9 ± 22.75	

Ferroni (2019)	Retrospective	LIAP vs. HIAP	6 vs. 15	300/300	29.8 ± 6.2	62.1 ± 9.75	53.4 ± 47.5	8.1 ± 20.13
					29.4 ± 7.25	62.2 ± 9	52.7 ± 29.75	7.5 ± 8.25

Christensen (2016)	Retrospective	LIAP vs. HIAP	12 vs. 15	100/100	29.88 ± 6.5	63.6 ± 8.25	NA	NA
					29.38 ± 7.25	62.04 ± 8		

Modi (2015)	Retrospective	LIAP vs. HIAP	15 vs. 20	201/550	NA	58.7 ± 6.5	49.1 + 17.5	5.97 + 3.60
						59.7 ± 7.0	48.3 + 20.0	6.44 + 7.69

BMI, body mass index; LIAP, low intraabdominal pressure; HIAP, high intraabdominal pressure; *X* ± *Y*, mean ± standard; NA, not available; NR, not report.

**Table 2:** The demographics of the studies.

Outcome	Variable	Model	MD (95% CI)	*P* value	*I* ^2^ (%)
Postoperative ileus	Age	Fixed	−0.40 (−1.22, 0.41)	0.33	53
	BMI	Fixed	0.36 (−0.41, 1.13)	0.36	0
	Prostate weight	Fixed	0.81 (−1.85, 3.47)	0.55	0

Hematoma	Age	Fixed	−0.48 (−1.38, 0.43)	0.30	52
	BMI	Fixed	0.36 (−0.41, 1.13)	0.36	0
	Prostate weight	Fixed	0.78 (−1.89, 3.45)	0.57	0

Positive margin rate	Age	Fixed	0.41 (−0.84, 1.66)	0.52	31
	BMI	Fixed	0.36 (−0.41, 1.13)	0.36	0
	Prostate weight	Fixed	0.86 (−4.10, 5.81)	0.73	0

Urinary retention	Age	Fixed	−0.40 (−1.22, 0.41)	0.33	53
	BMI	Fixed	0.42 (−0.52, 1.36)	0.38	0
	Prostate weight	Fixed	0.78 (−1.89, 3.45)	0.57	0

Operative time	Age	Fixed	−0.40 (−1.22, 0.41)	0.33	53
	BMI	Fixed	0.42 (−0.52, 1.36)	0.38	0
	Prostate weight	Fixed	0.84 (−1.68, 3.36)	0.51	0

Intraoperative blood loss	Age	Fixed	−0.40 (−1.22, 0.41)	0.33	53
	BMI	Fixed	0.42 (−0.52, 1.36)	0.38	0
	Prostate weight	Fixed	0.84 (−1.68, 3.36)	0.51	0

BMI, body mass index.

## Data Availability

The original contributions presented in the study are included in the article material. Further inquiries can be directed to the corresponding author.

## References

[1] Pasticier G., Rietbergen J. B., Guillonneau B., Fromont G., Menon M., Vallancien G. (2001). Robotically assisted laparoscopic radical prostatectomy: feasibility study in men. *European Urology*.

[2] Lowrance W. T., Eastham J. A., Savage C. (2012). Contemporary open and robotic radical prostatectomy practice patterns among urologists in the United States. *The Journal of Urology*.

[3] Checcucci E., Amparore D., De Luca S., Autorino R., Fiori C., Porpiglia F. (2019). Precision prostate cancer surgery: an overview of new technologies and techniques. *Minerva Urologica e Nefrologica*.

[4] Hu J. C., Gu X., Lipsitz S. R. (2009). Comparative effectiveness of minimally invasive vs. open radical prostatectomy. *JAMA*.

[5] Srougi V., Sanchez-Salas R. (2017). Re: robot-assisted laparoscopic prostatectomy versus open radical retropubic prostatectomy: early outcomes from a randomised controlled phase 3 study. *European Urology*.

[6] Zhong W., Roberts M. J., Saad J. (2020). A systematic review and meta-analysis of pelvic drain insertion after robot-assisted radical prostatectomy. *Journal of Endourology*.

[7] Neudecker J., Sauerland S., Neugebauer E. (2002). The European Association for Endoscopic Surgery clinical practice guideline on the pneumoperitoneum for laparoscopic surgery. *Surgical Endoscopy*.

[8] Borg I. R. A. M. M. Z., Lim A., Verbrugge S. J. C., Ijzermans J. N. M., Klein J. (2004). Effect of intraabdominal pressure elevation and positioning on hemodynamic reponses during carbon dioxide pneumoperitoneum for laparoscopic donor nephrectomy: a prospective controlled clinical study. *Surgical Endoscopy*.

[9] Ploussard G. (2018). Robotic surgery in urology: facts and reality. What are the real advantages of robotic approaches for prostate cancer patients?. *Current Opinion in Urology*.

[10] Cullen D. J., Coyle J. P., Teplick R., Long M. C. (1989). Cardiovascular, pulmonary, and renal effects of massively increased intra-abdominal pressure in critically ill patients. *Critical Care Medicine*.

[11] Kashtan J., Green J. F., Parsons E. Q., Holcroft J. W. (1981). Hemodynamic effects of increased abdominal pressure. *Journal of Surgical Research*.

[12] Mutoh T., Lamm W. J., Embree L. J., Hildebrandt J., Albert R. K. (1991). Abdominal distension alters regional pleural pressures and chest wall mechanics in pigs in vivo. *Journal of Applied Physiology*.

[13] Ost M. C., Tan B. J., Lee B. R. (2005). Urological laparoscopy: basic physiological considerations and immunological consequences. *The Journal of Urology*.

[14] Chiu A. W., Azadzoi K. M., Hatzichristou D. G., Siroky M. B., Krane R. J., Babayan R. K. (1994). Effects of intra-abdominal pressure on renal tissue perfusion during laparoscopy. *Journal of Endourology*.

[15] Hua J., Gong J., Yao L., Zhou B., Song Z. (2014). Low-pressure versus standard-pressure pneumoperitoneum for laparoscopic cholecystectomy: a systematic review and meta-analysis. *The American Journal of Surgery*.

[16] Rohloff M., Cicic A., Christensen C., Maatman T. K., Lindberg J., Maatman T. J. (2019). Reduction in postoperative ileus rates utilizing lower pressure pneumoperitoneum in robotic-assisted radical prostatectomy. *Journal of Robotic Surgery*.

[17] Rohloff M., Peifer G., Shakuri-Rad J., Maatman T. J. (2021). The impact of low pressure pneumoperitoneum in robotic assisted radical prostatectomy: a prospective, randomized, double blinded trial. *World Journal of Urology*.

[18] Hampson A., Raj N., Lingamanaicker V. (2021). Serum cytokine levels as markers of paralytic ileus following robotic radical prostatectomy at different pneumoperitoneum pressures. *Current Urology*.

[19] Raval A. D., Deshpande S., Koufopoulou M. (2020). The impact of intra-abdominal pressure on perioperative outcomes in laparoscopic cholecystectomy: a systematic review and network meta-analysis of randomized controlled trials. *Surgical Endoscopy*.

[20] Higgins J. P. T., Altman D. G., Gotzsche P. C. (2011). The Cochrane Collaboration’s tool for assessing risk of bias in randomised trials. *BMJ*.

[21] Sterne J. A., Hernan M. A., Reeves B. C. (2016). ROBINS-I: a tool for assessing risk of bias in non-randomised studies of interventions. *BMJ*.

[22] Christensen C. R., Maatman T. K., Maatman T. J., Tran T. T. (2016). Examining clinical outcomes utilizing low-pressure pneumoperitoneum during robotic-assisted radical prostatectomy. *Journal of Robotic Surgery*.

[23] Modi P. K., Kwon Y. S., Patel N. (2015). Safety of robot-assisted radical prostatectomy with pneumoperitoneum of 20 mm Hg: a study of 751 patients. *Journal of Endourology*.

[24] Ferroni M. C., Abaza R. (2019). Feasibility of robot-assisted prostatectomy performed at ultra-low pneumoperitoneum pressure of 6 mmHg and comparison of clinical outcomes vs. standard pressure of 15 mmHg. *BJU International*.

[25] Johnstone C., Hammond J., Hanchanale V. (2021). Is the use of ultra-low insufflation pressure safe and feasible in robot assisted radical prostatectomy. *Türk Üroloji Dergisi/Turkish Journal of Urology*.

[26] Moschini M., Morlacco A., Kwon E., Rangel L. J., Karnes R. J. (2017). Treatment of M1a/M1b prostate cancer with or without radical prostatectomy at diagnosis. *Prostate Cancer and Prostatic Diseases*.

[27] Iyer S., Saunders W. B., Stemkowski S. (2009). Economic burden of postoperative ileus associated with colectomy in the United States. *Journal of Managed Care Pharmacy*.

[28] Schilling M. K., Redaelli C., Krahenbuhl L., Signer C., Buchler M. W. (1997). Splanchnic microcirculatory changes during CO2 laparoscopy. *Journal of the American College of Surgeons*.

[29] Hsu R. L., Kaye A. D., Urman R. D. (2013). Anesthetic challenges in robotic-assisted urologic surgery. *Reviews in Urology*.

